# Dual Optimized Sulfonated Polyethersulfone and Functionalized Multiwall Carbon Tube Based Composites High Fouling Resistance Membrane for Protein Separation

**DOI:** 10.3390/membranes12030329

**Published:** 2022-03-16

**Authors:** Muhammad Irfan, Masooma Irfan, Ani Idris, Abdullah Saad Alsubaie, Khaled H. Mahmoud, Noordin Mohd Yusof, Naeem Akhtar

**Affiliations:** 1 Centre for Environmental Sustainability and Water Security (IPASA), School of Chemical and Energy Engineering, Universiti Teknologi Malaysia, Johor Bahru 81310, Malaysia; irfanutm@gmail.com; 2 Interdisciplinary Research Centre in Biomedical Materials, Lahore Campus, COMSATS University Islamabad, Defense Road, Off Raiwind Road, Lahore 54000, Pakistan; naeemkhan@cuilahore.edu.pk; 3 Department of Chemistry, Lahore Campus, COMSATS University Islamabad, Defense Road, Off Raiwind Road, Lahore 54000, Pakistan; masooma.uthm@gmail.com; 4 Department of Physics, College of Khurma University College, Taif University, P.O. Box 11099, Taif 21944, Saudi Arabia; asubaie@tu.edu.sa (A.S.A.); k.hussein@tu.edu.sa (K.H.M.); 5 School of Mechanical Engineering, Faculty of Engineering, Universiti Teknologi Malaysia, Johor Bahru 81310, Malaysia; noordin@utm.my

**Keywords:** membrane, carbon nanotube, protein, antifouling

## Abstract

Commercial grade sulfonated-Polyethersulfone (S-PES) and functionalized multiwall carbon nanotube (f-MWCNT)/polyvinylpyrrolidone (PVP) nanocomposites (NCs) were used to enhance and optimize the antifouling, protein resistance and protein separation properties of the S-PES ultrafiltration membranes. The polarities of sulfonic groups of S-PES, carbonyl carbon of pyrrolidone, hydroxyl and carboxyl groups of f-MWCNT in the membrane composition helped to strongly bind each other through hydrogen bonding, as shown by Fourier-transform infrared spectroscopy (FTIR). These binding forces greatly reduced the leaching of NCs and developed long finger-like projection, as confirmed by elution ratio and cross-sectional studies of the membranes via field emission scanning electron microscope (FESEM). The contact angle was reduced up to 48% more than pristine PES. Atomic force microscopy (AFM) was employed to study the various parameters of surface roughness with 3d diagrams, while grain analysis of membrane surface provided a quantitative estimation about volume, area, perimeter, length, radius and diameter. The NCs/S-PES enhanced the flux rate with an impressive (80–84%) flux recovery ratio and (58–62%) reversible resistance (*R_r_*) value in situ, with 60% and 54.4% lesser dynamic and static protein adsorption. The best performing membrane were reported to remove 31.8%, 66.3%, 83.6% and 99.9% for lysozyme-(14.6 kDa), trypsin-(20 kDa), pepsin-(34.6 kDa) and bovine serum albumin (BSA-66 kDa), respectively.

## 1. Introduction

Protein purification strategies are currently being employed in industrial and academic applications, with particular emphasis on methodologies, which are implemented for the production of recombinant proteins of biopharmaceutical importance. Therefore, protein concentration and purification is one of the most intensive separation processes for biotechnology, biomedicine and food production industries [1]. Nowadays, several separation approaches have been applied to separate and purify proteins from its mixture. These include adsorption, electrophoresis membrane contactors, ultrafiltration (UF), tangential flow filtration and chromatography [2,3,4,5].

UF, in particular, has been gaining much more consideration because it is easy to handle, does not require chemical additives and could be easily scaled up at a low operational cost. Furthermore, it is more efficient to other clearance methods [6,7,8,9].

The main component of a membrane filtration process is the property of a membrane that could offer high separation efficiency against proteins without experiencing severe fouling. The phase inversion method is commonly used to fabricate the UF membranes sold in the market. Mostly, these membranes are made of hydrophobic/semi-hydrophobic polymers such as polyethersulfone (PES), polyvinylidene fluoride, polypropylene and Polysulfone [10,11,12,13].

PES is a highly thermoplastic, relatively less flammable and chemically resistant polymer. It is, however, relatively hydrophobic in nature and has less water sorption. It will cause the PES membrane to have a strong interaction with proteins via hydrophobic and electrostatic forces during the protein filtration. These, as a result, negatively affect the membrane water flux and increase membrane fouling, leading to frequent membrane cleaning. For that reason, the PES-based membrane is frequently modified to increase its anti-fouling propensity before its practical use [14,15].

In the area of nanotechnology, polymer matrix-based nanocomposites have generated a significant amount of attention in the recent literature. Inorganic nanoparticles were used as fillers to form nanocomposite membranes due to their hydrophilicity, large specific surface area, pore channels and other functional characters. There are many inorganic nanoparticles, such as silica, Al_2_O_3_, Fe_3_O_4_, ZnO, and CdS, ZrO_2_, TiO_2_, zeolite, and carbon nanotubes that are used in the fabrication of nanocomposite membranes with improved antifouling properties. This type of membrane has brought a new concept to improving membrane separation efficiency during the water treatment process, but it is not the main research focus of studies on the protein separation process [10,16,17,18].

The blending of PES with hydrophilic nanomaterials attracts much interest due to its ability to improve the permeate flux and rejection. Hydrophilic nanomaterials are responsible for enhancing water absorption during a filtration process, which reduces water transport resistance and improves membrane permeability. Multiwall carbon nanotubes (MWCNTs) were previously used as inorganic fillers to increase membrane performance because of its exceptional chemical, mechanical and electrical properties. However, its hydrophobic and inert nature tends to have dispersion difficulty in some solvents. In order to fully disperse the MWCNTs, different modification methods are used, among which acid treatment is the best to perform at the laboratory and industrial levels. The acid treatment of MWCNT could cause oxidation on the nanotube surface, improving its hydrophilicity. With the improvement in hydrophilicity, MWCNTs tend to have the better dispersion capability in solvent [19,20]. Phao et al. [21], for instance, improved the wettability and dispersion ability of CNTs by doping them with nitrogen. The modified CNTs were then incorporated into the PES dope solution to fabricate a new type of membrane exhibited 70% flux improvement compared to the control PES membrane (without CNTs incorporation).

Besides inorganic hydrophilic nanomaterials, hydrophilic polymers, such as PVP, are also always considered as an additive to improve the hydrophilicity of the PES-based membrane. PVP is a highly polar, physiologically inert, non-ionic, amphiphilic and water-soluble polymer. It swells in aqueous media and, thus, can be used to modify membrane performance. PVP has been previously reported to have a positive influence in improving MWCNT dispersion quality in different solvents [6,7].

Functionalization of the polymer backbone is a technique to improve the hydrophilicity without compromising membrane separation performance. Sulfonation of polymer, for instance, could increase not only membrane hydrophilicity for better antifouling properties, but also increase membrane permeability. Sulfonated polymers were also reported to have greater thermal, chemical, mechanical and separation properties on the resultant asymmetric membranes. Irfan et al., modified the PES membrane with S-PES to enhance the permeability, pore size, hydrophilicity and sub-layer porosity of the membrane [15]. Wang et al. successfully minimized protein adsorption using the PES membrane blended with S-PES [22]. Since sulfonation increases the anionic character on the membrane surface, the developed membrane tends to have greater electrostatic repulsion towards protein molecules and favor reduced fouling phenomena [14]. Use of S-PES polymer is a good choice for antifouling membranes, but its preparation at the lab scale seems quite difficult because of the very sensitive preparation parameters, as reported by Guan et al., and Deing et al. [23,24].

**Figure 1:**
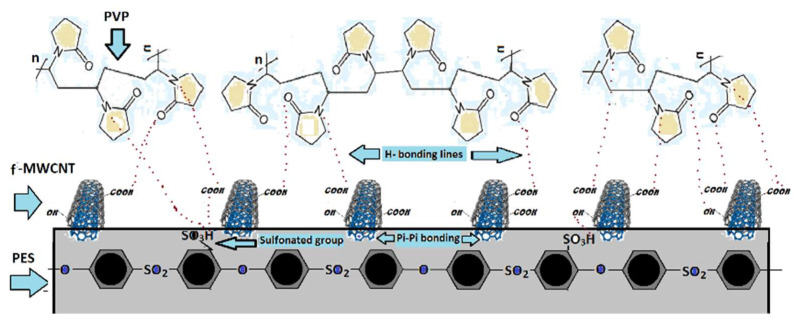
Schematic representation of SPES and NCs (f-MWCNT/PVP) arrangement in fabricated membranes.

In the current research work, membrane performances were optimized using two-step process; one of them is addition blending to the casting solution and other is immobilization of polymer with hydrophilic fragments. In the first step, oxidized MWCNTs were prepared via the treatment of pristine MWCNT with a mixture of concentrated nitric and sulfuric acids and then mixed vigorously PVP to produced NCs by hydrogen bonding. In the second step industrial grade S-PES with higher molecular weight were blended with PES polymer and f-MWCNT/PVP-based NCs to produce a new type of UF membrane that could exhibit greater surface hydrophilicity and antifouling resistance. Figure 1 represents the schematic representation of the possible structure of S-PES/NCs-based membranes. The effects of S-PES/NCs on the PES-based membranes were studied by FESEM, contact angle, and surface free energy. Detailed AFM analyses were performed to observe the surface roughness, pore profile and 3D images. Moreover, experiments that include static and dynamic BSA adsorption, membranes antifouling performances and separation performance were also conducted and discussed in detail.

## 2. Materials and Methods

The S-PES polymer having molecular weight 90,000 Dalton and 36% sulfonation degrees was purchased by Konish chemical ind. Co., Ltd (Wakayama, Japan). Polyvinylpyrrolidone-K90 and BSA (67K Da) were purchased from Fluka, dimethyl formamide (average molecular weight = 80.14 g/mole), pepsin (35 KDa), trypsin (20 KDa) and lysozyme (14.6 KDa) were obtained from Sigma-Aldrich. The pristine MWCNT (length: 12 micron, Average dia: 10 nm) was provided by the company of Chengdong (Shenzhen, China).

### 2.1. Acid Treatment MWCNT, NCs and Membrane Formation

The oxidation of MWCNTs with the mixture of concentrated nitric and sulfuric acids produced the –COOH and –OH functional groups. These functional groups impart the polarity to the nanotube layer. In addition, such a treatment resulted in a removal of the metallic impurities [25]. In brief, raw MWCNTs were blended with a mixture of concentrated nitric and sulfuric acid for 24 h at 110 °C, followed by filtration (using 0.22 um polycarbonated membrane) and washing with distilled water until the pH ≅ 6–7.

Two-step processes were used for preparation of dope solutions. In the first step, different types of NCs (Table 1, Step 1) were produced by blending functionalized MWCNTs (f-MWCNTs) with PVP-K90 in DMF for 6 h at high stirring speed. In the second step, NCs mixture was transferred into a round bottom flask attached to the conventional heating mantle, followed by the addition of PES and S-PES polymer as per the formulation shown Table 1. The final homogenous dope solutions were allowed to cool down before storing in the glass bottles. Flat sheet membranes (160–200 um thickness) were cast using a casting knife on a clean glass plate by dry-wet phase inversion method. Distilled water was used as a coagulation bath at room temperature. After the post treatment in hot water bath, the membranes were ready for further evaluation.

In Table 1, membranes are named in ascending order of S-PES amount, its mean notations 15, 30, and 45 in membrane name like M1-15, M2-30 and M3-45 showed weight percentage of S-PES in correspond to total weight of polymer.

### 2.2. Characterizations

#### 2.2.1. FTIR, XRD and FESEM

The instrument’s spectrum “One B, Perkin-Elmer” was used to determine FTIR spectrums of the MWCNT, f-MWCNTs, S-PES and the bonding chemistry of NCs and its corresponding membranes. Prior to analysis, all the samples were heated at 70 °C for 2 h to minimize the possible effect of moisture. The XRD patterns of the raw MWCNT and acid treated MWCNT (f-MWCNT) were studied by the D8 Advance X-ray, Bruker instrument. The JEOL JSM-7500F was used to determine the cross-sectional morphology of all formulated membranes. Prior to this analysis, the membrane samples were prepared in liquid nitrogen, then by sputter-coated with platinum.

#### 2.2.2. Hydrophilicity

Surface hydrophilicity of the fabricated membranes were determined by the sessile-drop dynamic method (CAM 101 optical Contact Angle Meter, KSV Instruments, Helsinki, Finland) using an optical contact angle measurement system.

#### 2.2.3. Surface Roughness Parameters via AFM

The Park XE-100 instrument of the AFM technique was used for generating 3D images of membrane top surface together with the its roughness parameters. The region tab of AFM software was used to determine the surface roughness parameters of the entire image, having a size (10 μm × 10 μm) [26]. The surface roughness parameters included were Min, Max, Mid and *R_pv_*, which symbolize the minimum height, maximum height, the average between the minimum and maximum height and peak-to-valley, respectively. *R_q_*, *R_a_*, *R_z_*, *R_sk_* and *R_ku_* that correspond to root-mean-squared roughness, roughness average, ten points average roughness, skewness and kurtosis of the line, respectively, were also included in this study.

#### 2.2.4. Surface Profiles via Grain Analysis

The grain analysis of the membrane surface proves to be a good technique to determine the various parameters of its surface profile, which include the volume, length, area, perimeter, radius and diameter of the detected grains. The XEI-AFM standard image processing and analysis software automatically provide the quantitative estimation of surface profiles parameters of scanned images [6].

#### 2.2.5. Porosity, Flux Rate and Flux Recovery

The flux rate *J* (*J_w_*_1_, *J_w_*_2_ and *J_w_*_3_) of all formulated membranes was measured using DI water and the protein solution *J_p_* (*J_p_*_1_ and *J_p_*_2_) at 3 bar pressure. The concentration of protein solution was kept at 1000 ppm and cross flow cell (effective membrane surface area of 42 cm^2^) was used for the experiments, whereas the Equation (1) was utilized for flux determination,

(1) $$J=\frac{V}{t\times A}\;$$

where, *V* is the volume of permeate (L), *t* is the time (h) and *A* is the effective membrane area (m^2^). The flux recovery was checked up to two cycles after fouling the test membranes with BSA solution (detail mention in Section 2.2.6) and Equations (2) and (3) was used to measure the flux recovery percentage (*R_FR_*) [27].

(2) $$For\;first\;cycle\;R_{FR1}\left(\%\right)=\frac{J_{w2}}{j_{w1}}\times 100$$

(3) $$\;For\;2nd\;cycle\;R_{FR2}\left(\%\right)=\frac{J_{w3}}{j_{w1}}\times 100$$

The water absorption experiments were also used to measure the porosity (ε) of all membranes and Equation (4) was used for this purpose. Unit of porosity lies between 0 and 100, represents percentage of pore space (p.u) in unit volume.

(4) $$\varepsilon \;\left(p.u\right)=\frac{w_{Wet}-w_{Dry}}{V.\;\delta_{w}}\;$$

where, $w_{Dry}$ and $w_{Wet}$ are the weights in gram of dry and wet membranes, *δ_w_* represents the density (0.998 g/cm^3^) of water and *V* is the pure water flux (mL).

The surface free energy (SL) of interaction at the interface between the liquid and the membrane surface (−ΔSL) was calculated using Young–Dupre revised equation (Equation (5)) [28],

(5) $$-\Delta \mathrm{SL}=\left(1-\cos\theta \right)\gamma_{L\;}^{T}\;$$

where, θ is the contact angle and $\gamma_{L\;}^{T}\;$is the total surface tension of water (72.8 mJ·m^−2^).

#### 2.2.6. Anti-Fouling and Membrane Resistances

The membrane fouling is of two types, one is the reversible fouling that can be removed by washing with DI water and second is the irreversible fouling that needs chemical cleaning agent. In this work, the antifouling property with respect to membrane resistance parameters were measured against BSA solution for up to two cycles. For the experiments, first *J_w_*_1_ was measured (a detail mentioned in Section 2.2.5) as the pure water flux for 1 h and then water was replaced with 1000 ppm BSA solution and its flux was noted as ‘*J_p_*_1_’ for the next 30 min under the same condition. After that, the membrane was cleaned with DI water and pure water flux was again measured as *J_w_*_2_ for the next 1 h. The test was continued (second cycle) and again water was replaced by the protein solution (*J_p_*_2_) followed by washing and measurement of flux as *J_w_*_3_.

For detailed analysis, the membrane total resistance rate (*R_t_*), the reversible resistance (*R_r_*) and the irreversible resistance (*R_ir_*) rates were calculated using Equations (6)–(8) for cycle one and Equations (9)–(11) for the second cycle of the membrane fouling, respectively [6].

(6) $$R_{t1}\left(\%\right)=\left(1-\frac{J_{p1}}{J_{w1}}\;\right)\times 100\;$$

(7) $$R_{r1}\left(\%\right)=\frac{J_{w2}-J_{p1}}{J_{w1}}\times 100\;$$

(8) $$\;R_{ir1}\left(\%\right)=\frac{J_{w1}-J_{w2}}{J_{w1}}\times 100\;$$

(9) $$R_{t2}\left(\%\right)=\left(1-\frac{J_{p3}}{J_{w1}}\;\right)\times 100\;$$

(10) $$R_{r2}\left(\%\right)=\frac{J_{w3}-J_{p3}}{J_{w1}}\times 100\;$$

(11) $$\;R_{ir2}\left(\%\right)=\frac{J_{w1}-J_{w3}}{J_{w1}}\times 100\;$$

#### 2.2.7. Protein Adsorption Studies

The antifouling behavior of the formulated membranes was also observed by the dynamic protein adsorption experiments, which were performed to conclude the quantitative estimation of adsorbed amount of BSA on the membrane surface. Small pieces of membranes (0.5 cm × 5 cm, *n* = 5) were immersed into the vials containing 10 mL of protein (1 g/L) solution at pH ≅ 7. The vials were kept on the water shaker at 25 °C for 6 h. After that, the membranes were removed from the protein solutions and the protein concentration was determined from the change in concentration of the protein solution before and after the adsorption via calibration curve method [27].

#### 2.2.8. Protein Transmission

The protein separation experiments were conducted using four different types of proteins, namely, lysozyme-14.6 kDa, trpsin-20 kDa, pepsin-34.6 kDa and BSA-66 kDa. The cross-flow cell was used for this experiment and 250 mL protein solution of each protein type having 1000 ppm concentration was filled in the feed container (*C_f_*) one by one and UF experiment was carried out at 3 bar pressure. Permeate (*C_p_*) was collected after 30 min. The protein rejection *R* (%) was calculated using Equation (12).

(12) $$R=\left(1-\frac{C_{p}}{C_{f}}\;\right)\times 100\;$$

## 3. Results and Discussion

### 3.1. MWCNT Functionalization

**Figure 2:**
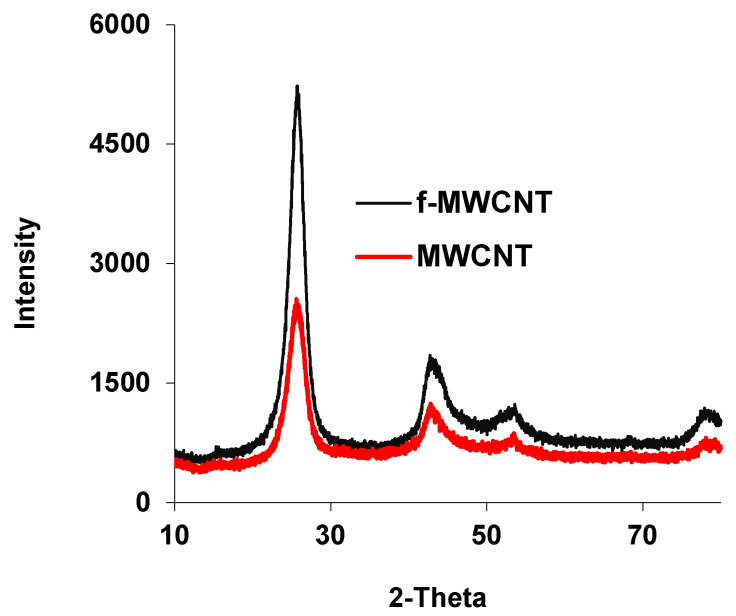
The XRD results of f-MWCNT and MWCNT.

**Figure 3:**
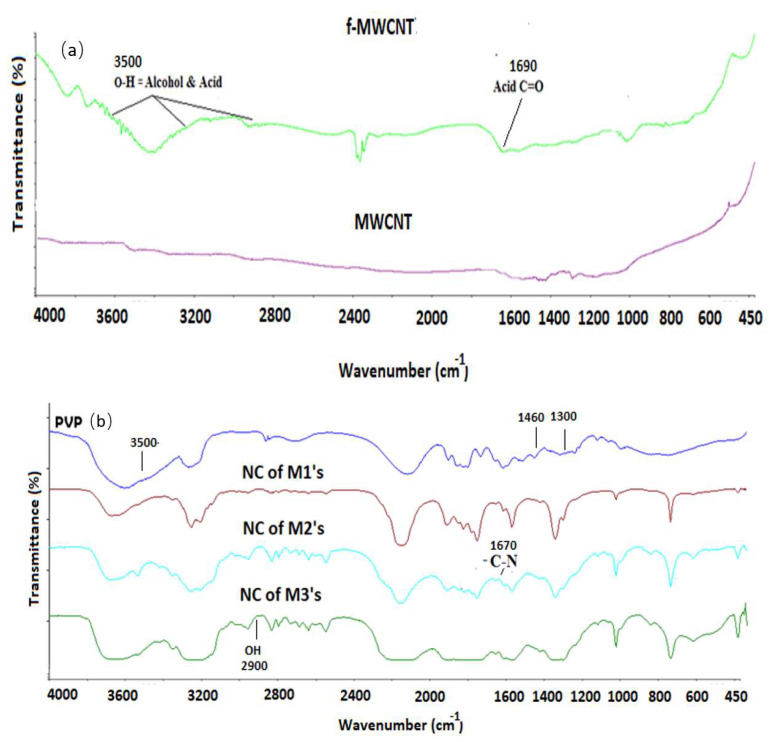
(**a**) FTIR spectra of MWCNT and f-MWCNT, (**b**) PVP and NCs.

The pristine MWCNTs are highly hydrophobic and generally exist in the form of bundles due to intrinsic van der Waals forces. They have very low solubility in solvent and limited interfacial bonding with polymers. Hence, homogeneously dispersed MWCNTs as well as good interfacial bonding in polymer-composites depend on the surface modification of the MWCNTs [29]. Acid treatment was carried out in this work to open ends of MWCNTs and to introduce carboxylic and hydroxyl groups on the MWCNTs surface via oxidation. Acid treatment was also found to be effective to minimize the π-π stacking or hydrophobic-hydrophobic interaction of the MWCNTs and reduce the extent of agglomeration [30]. The XRD spectra of MWCNT and f-MWCNT are shown in Figure 2, in which the f-MWCNTs exhibit the peak intensity up to the 5000 at 25.4° related to the (002) plane. The pristine MWCNT shows lower intensity of its XRD peak at (100) plane on the same angle than f-MWCNT. This observation is similar to the work of Buang et al. and Cheng et al., where higher peaks of f-MWCNT are related to the hydroxyl and carboxyl functional groups [31,32]. The FTIR analysis (Figure 3) is also used to confirm the presence of –COOH and –OH groups at the surface of f-MWCNTs. The comparison of MWCNT and f-MWCNT shows that the new peaks at 3500 cm^−1^ and 2900 are related to the hydroxyl group of alcohol and acid, while the peak at 1690 cm^−1^ indicates the presence of a carbonyl group of –COOH. Thus, the FTIR and XRD results verify the presence of new functional groups onto the f-MWCNT surface upon acid treatment process.

### 3.2. Nanocomposite and Corresponding Sulfonated Membranes (FTIR)

Figure 3 also shows the FTIR spectra of PVP and different types of f-MWCNT/PVP-based NCs. For the pure PVP, the vibrational bands at 1250, 1300, 1460, 1670 and 3500 cm^−1^ are assigned to the C–N stretching, CH_2_ wagging, C–H bending, amide group of PVP and pendent amide, respectively [7]. Whereas, for the NCs, the prominent peaks at 1670, 2900 and 3500 cm^−1^ are associated with the carbonyl group of the amide pyrrolidone ring and –OH group of alcohol/acids. The FTIR spectra can be used for the authentication of hydrogen bonding and the alteration of the FTIR band in terms of broadening or moved to a low absorption frequency range, related to H-bonding [33]. The comparison of NCs spectrum with the f-MWCNT and pure PVP shows that peaks of PVP at 1250, 1300 and 1460 cm^−1^ move to a slightly lower frequency range, whereas the vibrational band of f-MWCNT at 3600 cm^−1^ disappeared and the peak at 3500 cm^−1^ of pure PVP becomes shorter and broadened. Moreover, FTIR spectra from 1690 cm^−1^ (OH of f-MWCNT) to 1670 cm^−1^ (C=O, amide group of PVP) also show a prominent shift of peaks and spectrum peaks become broadened, disappear and move to lower frequency; this portion is highlighted in Figure 3. These changes confirm the presence of hydrogen bonding in all the NCs.

**Figure 4:**
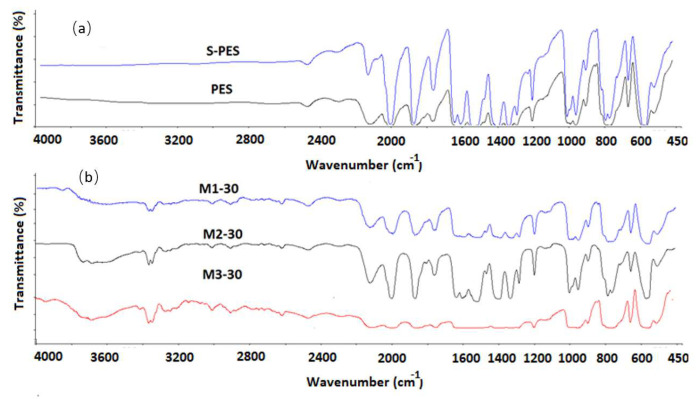
(**a**) FTIR spectra of S-PES and PES, (**b**) Nanocomposite membranes.

In this work, industrial grade S-PES polymer was used and the degree of sulfonation was verified by acid base titration scheme and by the FTIR spectrum. Figure 4 illustrates the FTIR results of PES, S-PES and some selected membranes. Compared to the FTIR spectrum of PES, the S-PES membrane shows a visible band at 1028 and 1187 cm^−1^ that belonged to the symmetric and asymmetric stretch of the sulfonate group, whereas the peak at 1134 cm^−1^ is correlated to the increment of –S=O groups [34]. For the PES, the peak at 1249 cm^−1^ is attributed to the asymmetric stretching vibrations of the S=O group, whereas aromatic rings of the PES show the bending vibration at 1480 and 1570 cm^−1^ [35]. In all the sulfonated/NCs membranes, the FTIR spectra at 3500 and 1650 cm^−1^ are changed, and peaks become broadened and shorter compared to the NCs spectrum (Figure 3). Moreover, the FTIR zone at 1000 to 1370 cm^−1^ displays the hydrogen bonding; FTIR line position is changed and almost broadened and straight, especially in the cases of M1-30 and M3-30 membranes. This area is co-related to –C–N, –SO_3_H and –S=O polar groups. Thus, the peaks at 3500, 1650 and 1050–1370 cm^−1^ confirm the presence of hydrogen bonding in the sulfonated/NCs membranes.

### 3.3. FESEM Analysis

**Figure 5:**
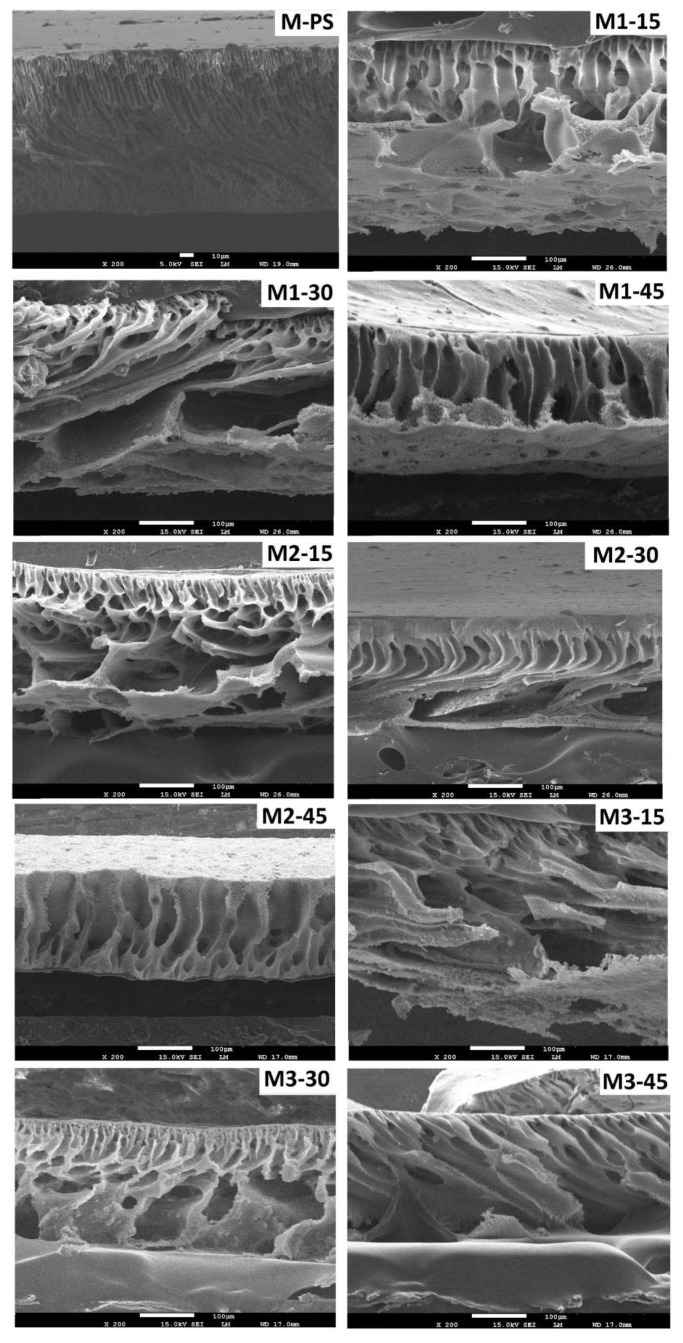
FESEM cross sectional images of PES membrane with and without the incorporation of NCs and S-PES.

The addition of NCs and S-PES could affect the phase separation kinetics of membrane formation, which ultimately change cross-sectional morphology of resulted membranes, as shown Figure 5.

The polar groups of NCs contribute the COO– and OH–, whereas S-PES provide –SO_3_H functional molecules that produce instability with non-polar groups of the polymer solution. This polar and non-polar combination in the dope solutions demonstrated the higher exchange rate between dope and coagulation bath liquid than non- polar composition of M-PS membranes. Thus, most of the S-PES/NCs-based membranes show typical asymmetric structure. The M-PS membrane, however, contains short finger-like entities and spongy structure. As the amount of sulfonation is increased from 15 to 45%, the shape of capillary changes. The M1-15 membrane demonstrates a typical dense layer and finger-like-entity that progressively transforms to open ends in M1-30, whereas the M1-45 membrane displays long channel-like structures. Similar trends are also found in M2 (M2-15 to M2-45) and M3 (M3-15 TO M3-45) series membranes, which are made of the same S-PES amount, although they contain a different amount of NCs. The findings suggest that sulfonated polymer is a dominant factor compared to NCs in affecting basic internal morphology of the membranes. It must be noted that the open-ended membranes generally lose their mechanical strength and are less practical for industrial applications [36].

### 3.4. Hydrophilicity and Elution Ratio

**Figure 6:**
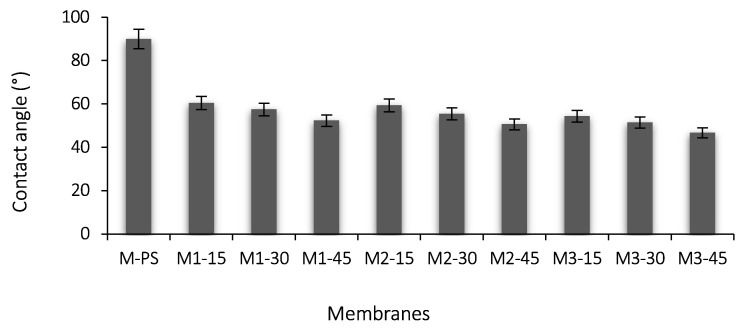
Comparison of PES membrane with and without NCs and SPES in terms of contact angle (*n* = 5).

The comparative results on membrane hydrophilicity are presented in Figure 6. It is observed that CA of M-PS is 89.88°, but the value significantly decreased to 46.75° upon the addition of S-PES/NCs additives in the M3-45 membrane. The membrane M1-15 shows a 32% reduction of CA compared to the M-PS that was further reduced by the increasing amount of S-PES polymer. Similar trends are observed from membrane M2-15 to M2-45 and M3-15 to M3-45. The membrane M3-45 displays a 48% greater reduction of CA compared to the pristine PES membrane. The low CA of M3-45 might be due to the higher amount of NCs and degree of sulfonation than all other formulated membranes. When the sulfonation degree increased from 15 to 30% and 45%, CA decreased by 3.3–4.5% and 5.2–5.7% in all membranes. These values indicate that the higher amount of S-PES in the dope solution enhanced the ionic effect on the membrane surface. Thus, the extent of S-PES looks directly proportional with the addition of hydrophilicity; these outcomes are parallel to the available works of Wang et al. [37].

**Figure 7:**
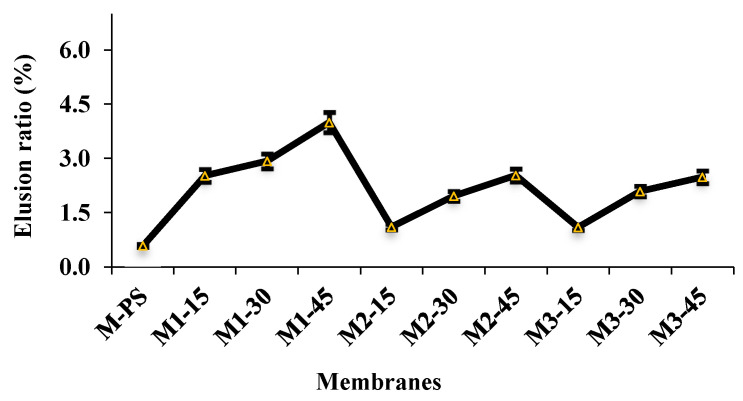
Elusion ratio of all formulated membranes (*n* = 5).

Figure 7 shows the quantitative measurements of the elution ratio of all the formulated membranes. All the membranes show some amount of elusion ratio. The M-PS membrane demonstrates the lowest elusion ratio (0.59%). This might be due to the loosening of some solvent molecules. The M1 series membranes (M1-15 to M1-45) show a higher amount of elusion than those of M2 and M3 series membranes. Moreover, as the sulfonation increases, the elution ratio also increases, a trend that is found in all membranes. The M1-45 membrane shows the highest value of elution (4%) compared to the M2-45 (2.54%) and M3-45 (2.49%) membranes, although it exhibits lower CA. It is observed that the increasing amount of f-MWCNT in the NCs from the M1 to M3 series membranes gradually reduces the elution ratio. In the NCs, the f-MWCNT has a dual-nature, i.e., hydrophobic (carbon-based chain) and hydrophilic (carboxylic and hydroxyl). Thus, it could act as a bridging material between PES and PVP due to pi-pi interaction and H-bonding. In the M1 series membranes, the amount of f-MWCNT is 0.1 wt.%, i.e., two and three times lower compared to M2 (0.2%) and M3 (0.3%) membranes, respectively. However, the 0.1% of f-MWCNT added is not able to hold the PVP and S-PES in the M1 membranes, mainly due to its high elusion ratio.

### 3.5. AFM Studies

#### 3.5.1. Surface Roughness Parameters

**Figure 8:**
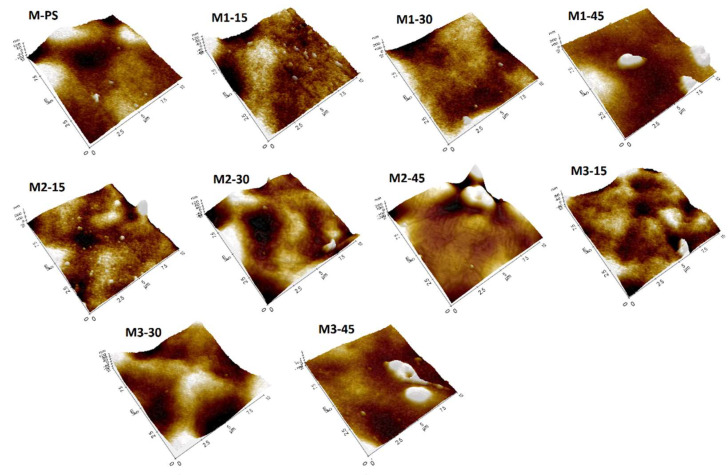
3D AMF pictures of membrane surface roughness of all formulated membranes.

The effects of S-PES and NCs on the membrane surface properties are further studied by AFM; the results are in Figure 8. The white and brown color represents the difference in surface heights on the membrane surface. The white spot shows the higher points on the membrane surface that might be due to the agglomeration of NCs. This agglomeration is found in the membranes that contained 45 wt.% S-PES in membrane composition. Since the S-PES has lower polymer weight than PES, the decrease in dope viscosity with increasing S-PES content is understandable [24]. The reduced viscosity is likely to cause less sheer stress on the NCs for its separation from its own agglomerates, leading to its poor dispersion and slight accumulation compared to other membranes. The quantitative measurements of image statistics with respect to surface roughness parameters are summarized in Table 2. The M1-15 shows a 123 nm maximum height of grains; the value is further increased to 275 nm in M1-30 and 289 nm in M1-45 membranes. A similar trend can also be found in the M2 series membranes. The findings show that the increasing load of S-PES polymer tends to enhance surface roughness. Rahimpour et al. [38] have also reported similar results in their work, where PES/S-PES blended membranes were prepared by the phase inversion method.

In the case of the M3 series membrane, the M3-45 shows the highest maximum heights (463 nm) compared to other formulated membranes. This suggests that as the S-PES polymer concentration increased in the dope, the distribution of NCs decreased, which was also visible in the 3D images of the membranes (Figure 8). Furthermore, the higher accumulation of NCs in M3-45 could be due to the excessive usage of f-MWCNT (0.3 wt.%) in the membrane, almost double that of M1 and M2 series membranes, which might require more dispersion power or time to achieve better dispersion. This work is in agreement with Daraei et al. [39], who reported that superior dispersion quality of CNTs was achievable when a low content of nanomaterials was added in the formulation. Parallel statements were conveyed by Vatanpour et al. [40], who blended MWCNT as an additive in PES-based membranes and improved the antifouling properties.

The dispersion effect of NCs seems to control the *R_a_* parameter of all the formulated membranes. The M-PS membrane shows 19.92 nm *R_a_* that was first reduced in all 15% S-PES-containing membranes. The *R_a_* value then progressively increases from 15 wt.% S-PES membranes to 45 wt.% S-PES containing membranes in all series. The increment of *R_a_* values with S-PES showed that the higher weight percentage of S-PES polymer favored the NCs aggregation.

#### 3.5.2. Grain Analysis (Surface Profile)

**Figure 9:**
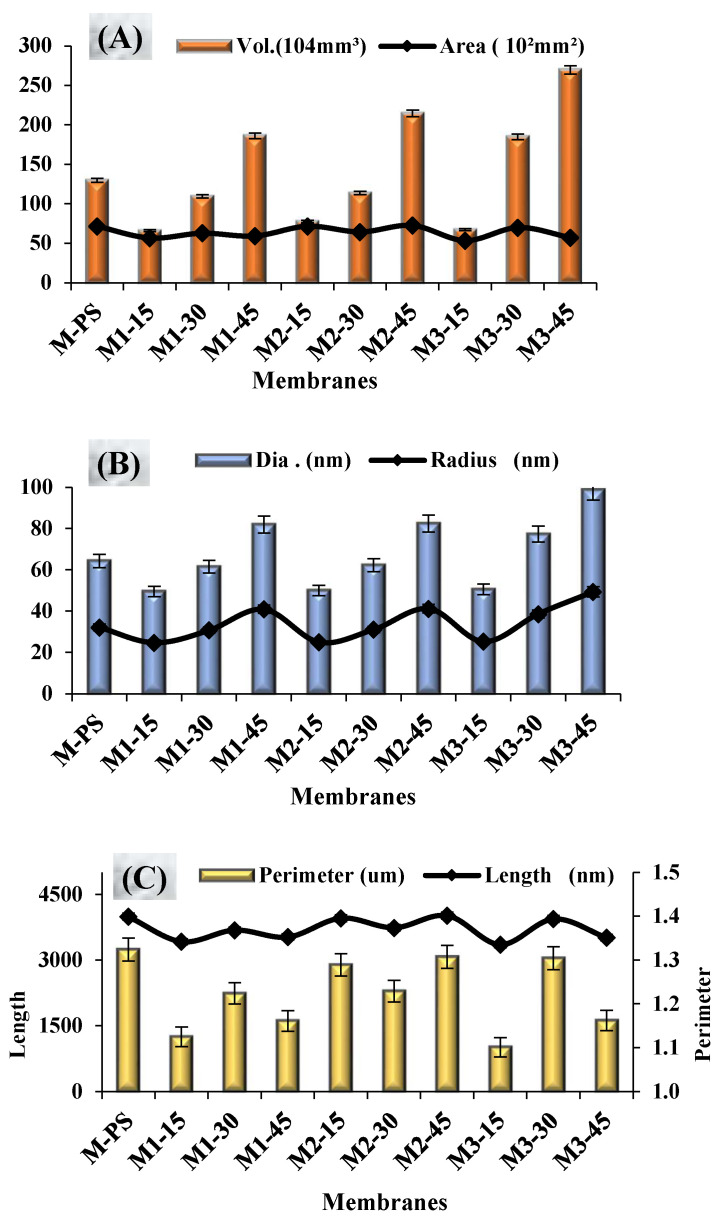
Surface Profiles result of all formulated membranes, obtained via XEI-AFM software of scanned images. (**A**)-The volume and area of grains, (**B**)-Radius and diameter of surface grain, (**C**)-Perimeter and length of grains.

The presence of S-PES and NCs in the dope formulation tends to improve the surface chemistry of PES-based membranes; the changes are analyzed by grain analysis graphs (Figure 9). Increasing the content of S-PES in the polymer solution from 15 to 45 wt.% increases the grain volume, radius and diameter of the resultant membranes in all membrane series (M1, M2 and M3), irrespective of the amount of NCs added (Figure 9A,B). The grain radius and diameter of M1 (M1-15 to M1-45) and M2 (M2-15 to M3-45) membrane are almost the same, but they exhibit different surface roughness (Table 2) and hydrophilicity (Figure 6). The observed results of perimeter, area and length of membrane grains show similar trends; these results are also supported by the FESEM images.

As mentioned earlier, in the FESEM images (Figure 5) of the membranes, the increased amount of S-PES from 15 to 45 wt.% affected the capillary system and changed the shape from typical dense layered-finger-like projection (15 wt.%-S-PES) to progressively open-ended (30 wt.%-S-PES) and then again close-ended with long channel-like structures (45 wt.%-S-PES). This transformation was especially visible in M1 and M3 membrane series; the data of perimeter, area and length represent the same outcome. In the Figure 9A,B, the quantitative values of perimeter, area and length were first increased by 30 wt.% S-PES (compared to corresponding 15 wt.% S-PES membranes) and then decreased with 45 wt.% S-PES (compared to 30 wt.% S-PES membrane).

Its mean open-ended capillaries of M1-30 and M2-45 occupied more space than other membranes. Whereas, in the M2-30 membrane showed a lower value of perimeter, area and length than M2-15 and M2-45 membranes, because its FESEM images also showed that its capillaries were first closed and had not changed into the open-ended projection directly. The variation of surface profile parameters might be due to difference of NCs, especially the amount of f-MWCNT (0.1 to 0.3%) and its distribution in the membrane matrix.

### 3.6. Porosity and Flux Rate

**Figure 10:**
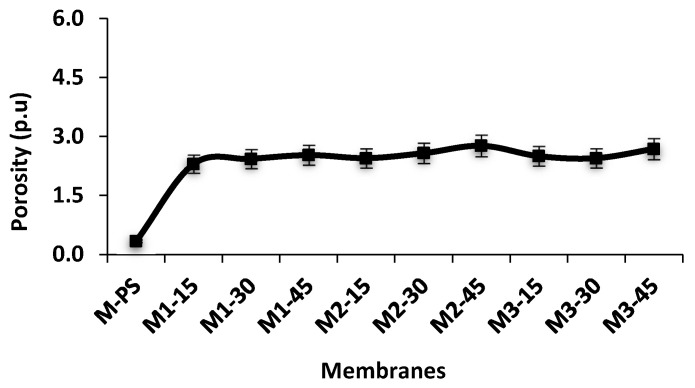

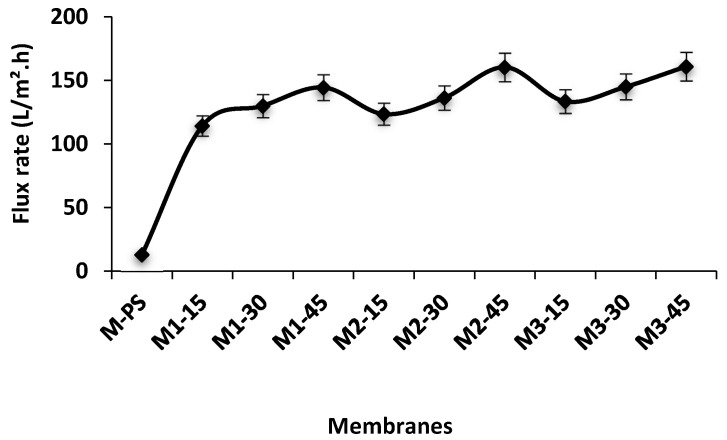
Pure water flux rate and porosity of all formulated membranes (*n* = 3).

Figure 10 presents the porosity and water flux of all fabricated membranes. As can be seen, incorporating S-PES and NC into the PES membrane matrix could enhance the porosity from 0.334 in M-PS to 2.77 in M2-45. The membranes from M1-15 to M1-45 and from M2-15 to M2-45 show progressive increment of porosity, but this increasing trend is not shown in the M3 series membranes. The M1 membranes show a lower porosity value than its corresponding membranes of the M2 series, suggesting that an increasing amount of NCs from 0.1 to 0.2% could positively improve the number of pores per unit area. The M3 membranes contain 0.3% of NCs and demonstrate a higher surface roughness owing to the accumulation of some NCs (Table 2). With respect to water flux, results show that all NCs/S-PES-based membranes exhibit higher flux rate than the pristine PES membrane. The flux rate of S-PES is 12.94 L/m^2^h, i.e., it increased 88.7% by the addition of NCs (0.1% f-MWCNT and 3% PVP) and 15% S-PES polymer. The flux rate is further increased in M1-30 and M1-45 membranes by the addition of 30% and 45% S-PES polymer. The M2-15 and M3-15 membranes show 89.6% and 90.4% higher flux rate compared to the M-PS membranes. The flux rates of M2 and M3 also increased with the addition of S-PES polymer; all three membranes series (M1, M2 and M3) show similar trends. The flux rates of M1-15, M2-15 and M3-15 are 114.4, 123.7 and 133.7 L/m^2^h, respectively. All these membranes contain 15 wt.% S-PES, but a variable amount of NCs. The increment of flux rate from M1 to M2 and M3 might be due to the amount of f-MWCNT added.

### 3.7. Antifouling Properties of Membranes

**Figure 11:**
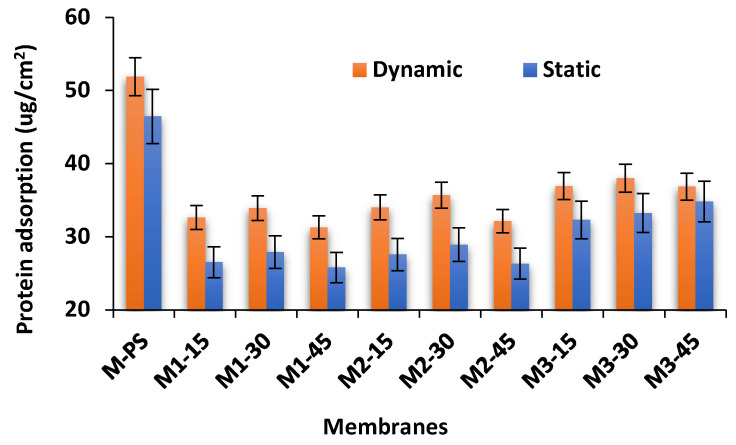
The static and dynamic protein adsorption results of fabricated membranes (*n* = 3).

Figure 11 presents the protein adsorption results of PES and S-PES/NC membranes. All membranes show lower adsorption of protein via the static method compared to the dynamic method. In the static method, the protein adsorption only occurs at the surface of the membranes, whereas, in the dynamic method, the stirring condition enforces the protein molecules to adsorb on the surface, as well as inside the membrane pores. The findings are in agreement with the work of Nakamura and Matsumoto [41], in which they also reported that protein adsorption is higher in the dynamic method compared to the static method. Moreover, it was found that all the S-PES/NCs membranes show lower protein adhesion than that of the M-PS membrane. According to Van der Bruggen et al. and Khulbe et al., the hydrophobicity of the PES membranes is its major weakness (severe fouling) for industrial application [42,43]. Liangliang et al. reported that higher protein adhesion on the membrane surface results from low hydrophilicity [44].

In this work, the higher hydrophobicity of M-PS membrane might be the reason for its higher protein adhesion in comparison to other formulated membranes. Since the hydrophilic membrane is preferable to adsorb water rather than solutes, it was also observed that lowering the contact angle results decreased the fouling of protein. All M3 series membranes show a higher amount of protein adsorption, which could be due to their greater surface roughness (see Table 2) compared to M1 and M2 membranes. The membranes that contain 45 wt.% S-PES show lower protein adsorption than those of membranes made of 15 and 30 wt.% S-PES. This indicates that higher amounts of sulfonation tend to create a repulsive effect against proteins and to reduce their adsorption.

**Figure 12:**
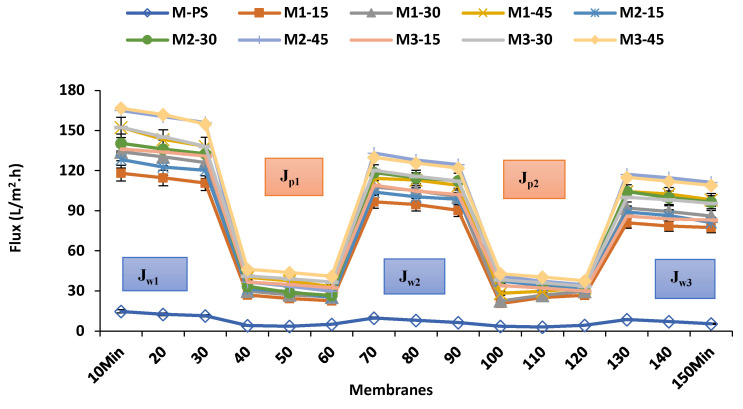
Initial pure water flux, BSA solution flux and after-washing flux for all prepared membranes.

Figure 12 compares the water flux profile of membranes before and after being used for the BSA UF process. The sharp decrement of the flux rate (*J*_*p*1_ and *J*_*p*2_) of the BSA solution is observed in all membranes, when pure water (feed solution) is replaced by the BSA solution. This reduction might be due to the accumulation and adsorption of protein molecules on the membrane surface and to inner pores that induce fouling.

**Figure 13:**
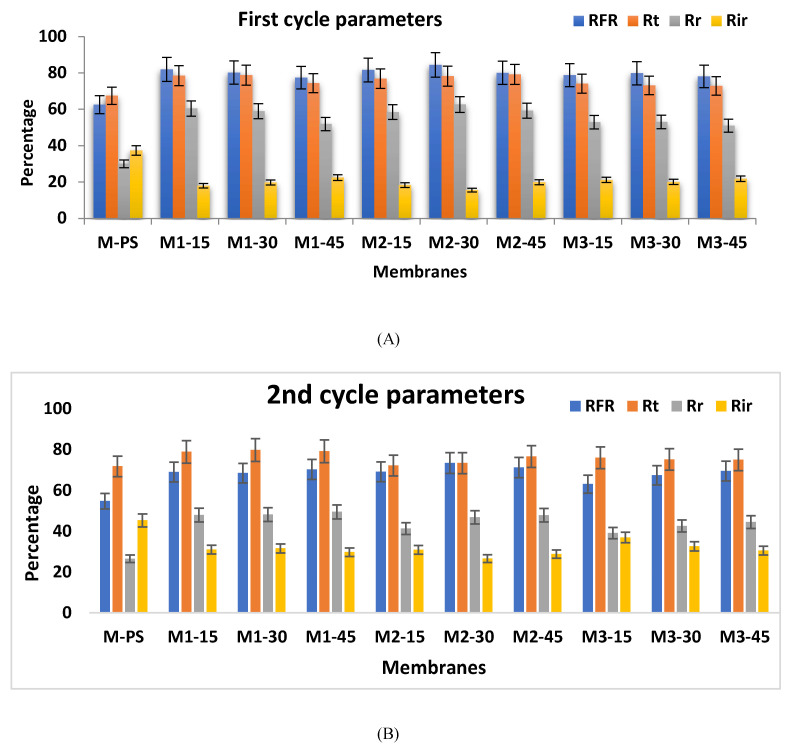
(**A**,**B**): Fouling parameters for all fabricated membranes, which included flux recovery (FFR), the membrane total resistance rate (*R_t_*), the reversible resistance (*R_r_*) and the irreversible resistance (*R_ir_*) rates (*n* = 3).

A more comparative and deeper assessment of antifouling may be obtained from the results shown in Figure 13A,B with respect to *R_FR_*, *R_t_*, *R_r_* and *R_ir_*; two round cycles of pure water and protein solution filtration were performed. As shown, M1-15 membrane records 82% FFR, 78.5% *R_t_* against BSA fouling, in which 60.5% are reversible. The M2-15 membrane demonstrates similar results compared to the M1-15 membrane, but with a slight reduction (3–4%) in *R_FR_* and *R_t_* compared to the M3-15 membrane. The slightly higher protein fouling of the M1-45 membrane could be attributed to its higher surface roughness. For the M1-15 to M1-45 membranes, they all show almost the same flux recovery and total resistance. It might be possible that, in the first round of water and BSA cycle (Figure 13A), the particular amount of fouling of protein molecules on the membrane surfaces eliminated the effect of surface roughness on the BSA solution in the 2nd round. Compared to other formulated membranes, the M2-30 membrane shows the highest value of FFR (84.41%), and 62.65% of the resistance is reversible. This higher value for the M2-30 membrane is persistent in the 2nd round of the BSA and water cycle. The M3-15 and M3-30 membranes, on the other hand, show lower antifouling resistance compared to their corresponding M1 (M1-15 and M2-15) and M2 (M2-15 and M2-30) series membranes. This might be due to the higher amount of NCs, especially considering that the 0.3 wt.% of f-MWCNT with 3 g of PVP was not shown to be good in the membrane formulation, leading to higher agglomeration of f-MWCNT and higher surface roughness, as discussed in Section 3.5.1.

### 3.8. Protein Separation

**Figure 14:**
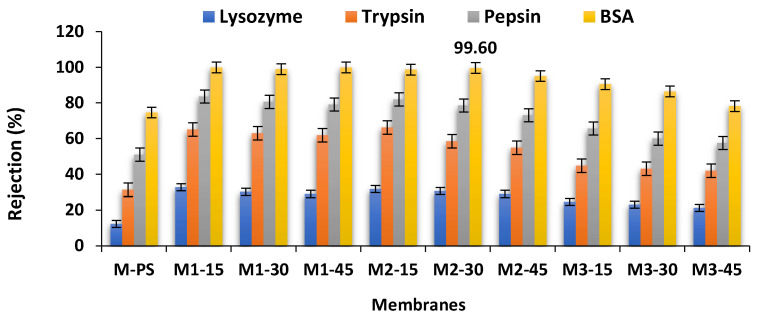
Separation performance of membranes in removing BSA, pepsin, trypsin and lysozyme using feed solution containing 1000 ppm solute (*n* = 3).

The filtration and separation efficiency of S-PES/NCs-based membranes were evaluated by the lysozyme, trpsin, pepsin and BSA proteins and Figure 14 shows the experiment results. All the sulfonated based membranes show a higher rejection rate of protein than non-sulfonated membranes (M-PES), although some of them possess higher grain diameters than the M-PS membranes. In all the membranes, the rejection rate of protein decreases with increased loadings of S-PES, irrespective of the amount of NCs added. The protein separation efficiencies of membranes are lower in the M1 series membranes than in the M2 and M3 series membranes. These results are consistent with protein adsorption and antifouling results, where the M1 series membranes show a less repulsive effect toward protein adhesion.

## 4. Conclusions

The membranes with improved anti-fouling properties were successfully prepared from the dope solutions composed of PES, S-PES and PVP/f-MWCNT-based NCs. The existence of S-PES and NCs in the PES-based membrane matrix was confirmed via FTIR. Except for the M-PS control membrane, other membranes exhibited asymmetric structure with long finger-like morphology. When the S-PES amount was increased from 15 to 45 wt.%, the effect of NCs seemed almost the same in all the membranes, and the weight percentage of S-PES in the membrane composition controlled the final orientation of the internal capillary system, hydrophilicity and grain analysis estimation. The NCs play an important role on the membrane surface roughness as well as the decrement of the leaching ratio of the hydrophilic component (PVP and S-PES). The blending of PES membrane with S-PES and NCs tended to improve membrane hydrophilicity, to increase surface roughness and to improve the membranes surface profile, as shown by AFM analysis. The protein adhesion studies indicated that the incorporation of the lesser amount of NCs and the higher quantity of S-PES would lead to lower protein adsorption and lower antifouling properties. The higher antifouling property of M2-30 membranes revealed that it has greater flux recovery compared to other formulated membranes. The M-PS control membrane had a very low flux rate with 63% *R_FR_* and 30% *R_r_*, whereas most of the NC/S-PES membranes demonstrated 80–84% *R_FR_* with 58–62% *R_r_*. The protein separation experiments showed that M1-15 to M1-45 membranes were able to reject 29–33% lysozyme, 62–65% trypsin, 79–83% pepsin, and almost 100% BSA, whereas other formulated membranes (M2 and M3 series) showed slightly lower separation efficiencies.

The current research work is limited to lab-scale evaluation, in which only three different percentages of SPES (15, 30 and 45) of total polymer weight were evaluated. The highest fouling resistance of 79% was achieved, with 38% of the resistance being irreversible, in the first fouling round, which can be further optimized. In terms of a future prospective, these membranes can be utilized on the industrial scale level and hollow fiber can be spun and evaluated using formulations of M1-15 to M1-45 membranes. These selected membranes demonstrated higher protein resistance with higher water flux.

## Figures and Tables

**Table 1:** Formulation of SPES and nanocomposite based membranes.

Formulations (Weight Percentage)						
Memb (M-CNT-SPES)	Step 1 (NCs)			Step 2		
	f-MWCNT	PVP	DMF	PES	S-PES	DMF
M-PS	-	-	-	16	-	84.00
M1-15	0.1	3	20	13.6	2.4	60.90
M1-30	0.1	3	20	11.2	4.8	60.90
M1-45	0.1	3	20	8.8	7.2	60.90
M2-15	0.2	3	20	13.6	2.4	60.80
M2-30	0.2	3	20	11.2	4.8	60.80
M2-45	0.2	3	20	8.8	7.2	60.80
M3-15	0.3	3	20	13.6	2.4	60.70
M3-30	0.3	3	20	11.2	4.8	60.70
M3-45	0.3	3	20	8.8	7.2	60.70

**Table 2:** The quantitative measurements of the image statistics from selected parts of the membranes represent the various parameters related to the surface roughness.

Membranes	Min (nm)	Max (nm)	Mid (nm)	*R_pv_* (nm)	*R_q_* (nm)	*R_a_* (nm)	*R_z_* (nm)	*R_sk_*	*R_ku_*
M-PS	−139.23	111.49	−13.87	250.72	26.82	19.92	242.18	−0.15	4.38
M1-15	−80.73	122.94	21.10	203.67	19.39	14.22	199.23	−0.42	5.98
M1-30	−87.55	275.02	93.74	362.57	25.23	18.81	355.73	−1.20	13.87
M1-45	−82.24	289.42	103.59	371.66	49.87	33.44	363.20	−2.13	8.97
M2-15	−60.41	117.85	28.72	178.26	16.03	12.01	173.69	−0.62	6.60
M2-30	−84.44	138.88	27.22	223.32	23.80	18.35	212.37	−0.23	3.89
M2-45	−245.73	280.15	17.21	525.88	47.41	31.44	516.70	0.19	6.61
M3-15	−84.79	297.59	106.40	382.39	21.66	14.63	377.02	−2.52	29.20
M3-30	−115.05	123.34	4.14	238.39	34.42	27.46	235.19	−0.11	3.04
M3-45	−125.77	462.56	168.40	588.34	74.13	50.15	581.85	−1.95	8.25
